# Letter to the Editor regarding “Brachial plexus compression following a laterjet procedure”

**DOI:** 10.1016/j.jpra.2025.08.025

**Published:** 2025-08-26

**Authors:** Olivier Camuzard, Tanguy Perraudin, Lucas Lo Cunsolo, Elise Lupon

**Affiliations:** a Department of Plastic and Reconstructive Surgery, Institut Universitaire Locomoteur et du Sport, University Côte d’Azur, Nice, France; b Université Côte d'Azur, CNRS, LP2M, France

**Keywords:** Latarjet procedure, Plexus brachial injury, Sural nerve graft, Anterior shoulder instability correction, Nerve reconstruction shoulder arthroscopy

Dear Sir,

With great interest, we read the manuscript written by Dounas et al.^1^ The authors reported an interesting case of brachial plexus compression caused by a residual pectoralis minor band that remained attached to the coracoid and was transposed to the anterior glenoid during a Latarjet procedure, leading to compression of the underlying cords. We would like to contribute additional insight by sharing our own experience with brachial plexus injury in the context of this procedure.

The Latarjet procedure is a well-established solution for the treatment of anterior shoulder instability. The authors present a case of a patient with an uncommon, subtle presentation of brachial plexus injury that fully resolved after neurolysis, which is valuable for clinicians to recognize.^1^ It is important to note, however, that different approaches to the Latarjet procedure exist, including both open and arthroscopic techniques.^2^ The authors did not explicitly specify whether the procedure in their case was performed using an open or arthroscopic Latarjet approach, although this distinction is important as it can significantly influence the type and severity of potential complications.^3^

The authors reviewed the literature on the mechanisms of brachial plexus injury associated with the Latarjet procedure and briefly mentioned iatrogenic lacerations without providing further details, an aspect that we would like to emphasize.^1^ Indeed, the arthroscopic procedure, as first described in our academic hospital,^4^ and now widely used worldwide, may lead to operative lacerations—an important risk that reconstructive surgeons should be aware of. This technique includes a delicate step of shaving the anterior glenoid rim, which carries a potential for severe neurovascular injury. We recently encountered such a complication in a 21-year-old right-handed student, a non-smoker and otherwise healthy, who presented with right anterior shoulder instability in the context of high-level sports activity. She underwent an arthroscopic Latarjet procedure that was complicated by an axillary vessel injury caused by the shaver, requiring emergency repair with a saphenous vein graft. She was referred to our reconstructive department 5 days later with a right infraclavicular brachial plexus injury, presenting with complete motor and sensory deficit of the right upper limb. We approached the brachial plexus through the deltopectoral groove and identified a complete transection of the lateral, medial, and posterior cords (Figure 1). The nerve gap was measured at 10 cm for the posterior cord and 8 cm for both the lateral and medial cords, using a second approach at the level of the brachial canal, which had already been explored during the arterial repair. Three end-to-end nerve grafts were performed using autologous sural nerve cable grafts matching the size of each defect, secured with 8–0 Prolene sutures to restore continuity of the three transected cords (Figure 2). The anastomoses were protected with fibrin sealant (TISSEEL®, Baxter Healthcare Corporation, Deerfield, IL, USA) and the limb was immobilized for 21 days.

**Figure 1:**
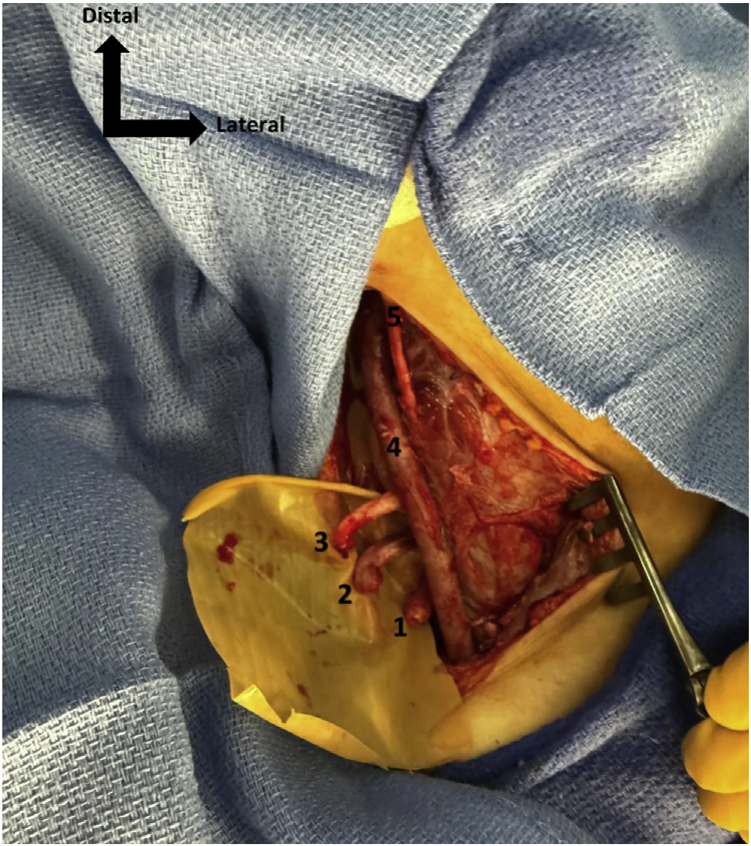
Distal stumps of the 3 cords of brachial plexus, externalized in the brachial canal. 1. Posterior cord, 2. Medial cord, 3. Lateral cord, 4. Venous bypass, 5. Musculocutaneous nerve.

**Figure 2:**
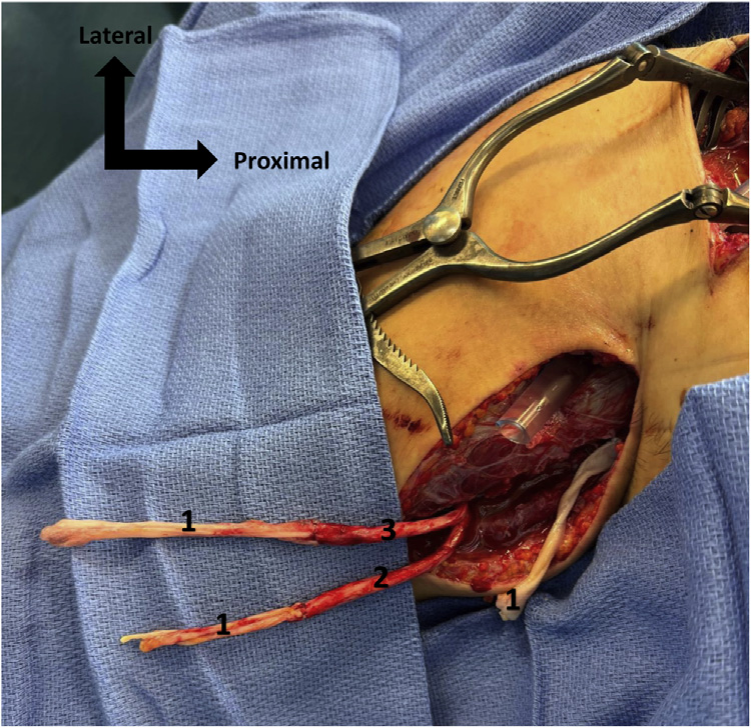
Sural nerve graft between distal stump of brachial plexus cords (already sutured) and proximal stumps of the 3 cords., 1. Sural nerve graft, 2. Medial cord, 3. Lateral cord.

Postoperative recovery was marked by severe pain requiring step 3 analgesics and regional anesthetic blocks for pain control. This case highlights an acute and devastating complication that may occur following arthroscopic Latarjet procedures, potentially jeopardizing limb viability. Given the extent of the nerve injuries, the prognosis for functional recovery remains guarded.^5^

We thank the authors for raising awareness of nerve injuries associated with this procedure and wish to emphasize another dramatic mechanism related to the shaving step of the surgery, along with a proposed surgical management strategy.

## Funding

This study has received no funding.

## Release of information

The study subject has consented to clinical photographs and imaging for academic research.

## Declaration of competing interest

The authors have no conflicts of interests to declare.
